# The Society for the Study of Diseases in Children

**Published:** 1906-05-26

**Authors:** 


### THE SOCIETY FOR THE STUDY OF DISEASES IN CHILDREN.

(The Wightman Lecture and Dinner in Honour of M. Broca, the Lecturer.)

About a hundred years ago England was accustomed to look to France for inspiration in medical matters. In those days, when facilities for foreign travel were immeasurably less than they are now, English medical men used to journey to Paris in order to profit by the instruction of their French *confrères.* The stirring period of the Napoleonic wars disturbed the even tenor of these visits, though it it said that some friendships formed between members of the profession on both sides of the Channel remained firm while racial animosity ran high. During the last few years, better understanding between the two nations on political questions has not been without its influence on professional feeling. Visits have been paid by French medical men to England and by English medical men to France.

A sequel of this interchange of visits has been the invitation of M. Broca, President of the Children's Disease Society in Paris, to deliver the Wightman lecture given annually before the kindred society in London. M. Broca, a distinguished son of a famous father, whose name is associated with that part of our brain which is of such pre-eminent importance to us all, took for his subject "Appendicitis in Children." The large number of cases that have been under his own care seems to suggest that this disease is more common in Paris than in London. M. Broca gave a review of all the varieties of this disease as it appears in children, and stated that some of the milder forms may give rise to repeated vomiting attacks which may closely simulate that curious affection, recurrent vomiting of children. M. Broca expressed the opinion that appendicitis is definitely a surgical disease, and that when the medical attendant is called to an acute case, within twenty-four hours from the onset of the symptoms, operation should be resorted to immediately. Should the case be first seen later than twenty-four hours from the onset, it requires to be dealt with according to the special indications it presents. If there are no indications that the peritoneum is seriously affected it is well to wait. If, however, the signs and symptoms are those of a general peritonitis, operation affords the patient the only chance, though it is well recognised that the hope of saving life is in any case very small. In conclusion, M. Broca referred to details of his method of operating for removal of the appendix and of evacuating localised collections of pus. At the close of the lecture a vote of thanks was proposed by Dr. Henry Ashby, of Manchester, and seconded by Dr. C. O. Hawthorne.

In the evening a dinner of the members of the Society for the Study of Disease in Children was held at the Imperial Restaurant, Mr. Clement Lucas occupying the chair. The health of the lecturer, M. Broca, was proposed by the Chairman, who said the Society tendered him their cordial thanks for the honour he had done them in coming so far and to a country which has acquired such a bad reputation for inclemency of weather, in order to give them some of the lessons he had learnt from his wide experience. M. Broca, in reply, said so far from finding this country inhospitable he had thoroughly enjoyed himself. Even the English Channel had dealt kindly with him, and the dinner prompted him to take the liberty of proposing a toast which was not on the programme. He felt sure that the dinner could do no harm since it had been blessed by the Archdeacon of Middlesex. He therefore begged out of gratitude to propose his health. The Archdeacon of Middlesex in a short speech thanked M. Broca for the honour so suddenly thrust upon him.

"The Society" was proposed by Sir W. H. Broadbent, who alluded to the great increase of medical societies which he had witnessed during his medical career. He thought such societies, by stimulating observation, benefited not only the profession but the public. He praised the organisation of the Society and the time at which the meetings are held; since it allows members living in the suburbs or even in the country, to attend them and return home the same evening.

Dr. Frederick Taylor and Dr. Henry Ashby responded on behalf of the Society. Dr. Taylor also spoke of the value of the Society and the mutual benefits derived from a connection with the provinces, where one of the meetings is held every year; while Dr. Henry Ashby expressed regret that diseases of children are almost entirely left out of the curriculum of medical students, and that they enter upon their life's work knowing next to nothing of this all-important branch. He thought that the examining bodies are largely responsible for this defective knowledge, and that possibly the secretaries of this Society might form a deputation to wait upon the Council of one of these august bodies and address them in words he believed attributed to Mr. Birrell: "Fellow-dunces, lend us your ears." Such a deputation might initiate some reform.

The health of the guests was proposed by Dr. W. Ewart and was responded to by Sir Thomas Barlow, who said that one of the originators of this Society had been spoken of as an innovator. He could not doubt that the innovation had been a valuable one and had already resulted in the production of much useful work.

